# Secondary metabolism is not secondary—why we need more cell biology in metabolomics

**DOI:** 10.1007/s00709-025-02104-7

**Published:** 2025-08-16

**Authors:** Peter Nick

**Affiliations:** Joseph Gottlieb Kölreuter Institute for Plant Sciences, Karlsruhe Institute of Technology, Karlsruhe, Germany

The distinction between primary and secondary metabolism was coined during a time, when the search for common principles of living cells gave rise to modern biology. Cell biology was the forerunner, but chemistry followed soon. In the attempt to define common lines of chemistry in living cells, Albrecht Kössel (1891) stated “*…muss auch die Chemie versuchen, diejenigen Bestandteile herauszusondern, welche in dem entwicklungsfähigen Protoplasma ohne Ausnahme vorhanden sind, und die zufälligen oder für das Leben nicht notwendigen Zellstoffe als solche zu erkennen*” (*also chemistry needs tostrive for sorting those compounds, which are found without exception in the developing protoplasm, from those that are random or not necessary for life*). For the metabolites shared by all cells, he proposed the term “primary”, while the specific compounds that were found only in some cells or only under certain conditions, appeared dispensible and, therefore, were baptised as secondary metabolites. Especially in plants and fungi, the term “secondary” seems inappropriate, though, because the diversity of these compounds often exceeds the wealth of “primary” metabolism. Moreover, “secondary” metabolism seems dispensible only, if one follows a reductionist viewpoint confined to individual cells. A broader scope shows clearly that, in plants, secondary metabolism comes first when survival of the plant as an entity is considered. Three contributions to the current issue highlight the central role of “secondary” metabolism.

In their contribution to the current issue, Sharma et al. (2025) comprehensively review the role of secondary metabolism for plant adaptation to the challenges of high altitude, focussing on the Himalaya. Especially elevated levels of UV-B, but also extreme shifts in temperature, challenge the redox homeostasis of these plants, mainly by interfering with photosynthetic electron flow across the thylakoids. The focus of their review is on the methylerythritol phosphate (MEP) pathway, giving rise to terpenoids, a group of compounds composed of isoprenic building blocks. These compounds are crucial for restoring redox homeostasis upon stress—on the one hand, mono- and sesquiterpenes, due to their conjugated double-bond system, are able to scavenge reactive oxygen species, and their volatility allows to act as systemic antioxidants. On the other hand, condensated polymers, such as gibberellins or abscisic acid, act as hormonal signals orchestrating defence against oxidative challenges. An interesting turn of their argument is a view on the medicinal effects of those compounds, explaining, why high-altitudes harbour such a wealth of medicinal plants. Thus, the potency of secondary metabolism not only safeguards these plants against a stressful environment, but also bears on human use of these plants.

Also, the contribution by Li et al. (2025) to the current issue deals with UV-tolerance, this time in alfalfa, an important fodder plant and a model organism for the economically relevant legumes. To improve UV-tolerance of crops, breeding for high levels of secondary compounds is a feasible strategy. However, this is a lengthy path, and it comes with costs, because the accumulation of secondary compounds binds resources that otherwise would be available for growth. Therefore, alternative approaches, where the plants are allowed to grow first and only then are treated by application of exogenous protectants, are of interest. The authors investigate the potential of melatonin in this regard. This derivative of tryptamine is known as hormonal signal in animals, for instance, favouring sleep in mammals. However, it also exists in plants and promotes UV-tolerance. The authors investigated the response of redox homeostasis under UV challenge after pre-treatment with either melatonin or its precursor 5-hydroxytryptamine, as well as the structurally similar, but inactive coumarin. They can demonstrate that the melatonine-treated plants cope better with UV-stress, and they show that this is linked with a lower level of reactive oxygen species. The underlying mechanism goes far beyond a direct radical scavenging of melatonin (due to its phenolic moiety, it is able to do so), but involves the activation of genes for enzymatic antioxidants. Interestingly, melatonin not only mitigates UV-stress, but recently has been found to render plants more tolerant to heat stress, again through improved redox homeostasis, can (Kolupaev et al. 2024). Thus, this “secondary” compound seems to be a central player, if one wants to render crop plants more resilient to the combined challenges of climate change.

From the viewpoint of medicinal use, alkaloids have attracted considerable attention. Many anti-tumour compounds including paclitaxel, vincristine, colchicine, or harringtonine belong to this chemically diverse group of secondary metabolites. Their synthesis is complex and often distributed across several cell types that team up to produce those compounds with their potent and highly specific bioactivity. Many alkaloids are also toxic for the plants themselves, creating the problem, how to generate them without self-damage. The solution to this conundrum is differentiation in space: the final steps of the pathway, culminating in the potent (and auto-toxic) final product, often proceed in terminally differentiating idioblasts, cells with impermeable barriers that shield their hazardous content from their neighbours and usually undergo programmed cell death during alkaloid processing. This sequestration is one of the reasons, why extraction of alkaloids often results only in tiny amounts of the desired product, because the content of the idioblasts is diluted by the content of the by far more abundant alkaloid-free cells. While the knowledge on the whereabouts of these idioblasts is crucial for pharmaceutical use, this knowledge is, surprisingly, lacking for numerous plants. In their contribution to the current issue, Richit and Kuhn (2025), search for alkaloid idioblasts in different clades of the Rutaceae, an important Angiosperm family, including relevant crops like Citrus. By means of classical histochemistry and anatomy, they can locate the root idioblasts in the root cortex and also extract knowledge about their chemistry. Since alkaloids are endowed with a ring-structure bearing a nitrogen, they can be solubilised with acid and washed out. Authors use this chemical property to design convincing negative controls for the specificity of their histochemical stains. This work not only gives a clear answer, where the alkaloids of this metabolically very active group of plants are located, it also shows, how useful classic histochemistry can be, if applied properly. Based on their findings, it is now possible to address the specific metabolic mechanisms needed for alkaloid biosynthesis—the corresponding genes will be active only in those idioblasts and would, therefore, gone unnoticed in the conventional approaches targeted on global changes in gene activity.

What did we learn from these three contributions? As a consequence of their photosynthetic lifestyle, plants need to extend their surface into the environment (contrasting with the strategy of animals that increase surface by involution to safeguard mobility). Developmental flexibility is a main survival strategy here, evident from their remarkable phenotypic plasticity. However, not as readily accessible to our eyes, this plasticity extends far beyond changes in shape and architecture: plant metabolism is highly flexible as well, and it is the “secondary” part of this metabolism that is most responsive to the environment and enables plants to overcome challenges posed by this environment. Given the importance of secondary metabolism, a central research task can be formulated: So far, the study of secondary metabolism is dominated by chemical approaches. Metabolomics looks for changes in abundance of metabolites irrespective of its localisation. However, this metabolism is often partitioned over different tissues, cell types, and, within a cell, different compartments. If we really want to understand the chemistry, we need to understand the spatial organisation of this chemistry. In other words: we need to bring cell biology into metabolomics. This is one of the missions, this journal stands for.
